# Assessing vaccine hesitancy using the WHO scale for caregivers of children under 3 years old in China

**DOI:** 10.3389/fpubh.2023.1090609

**Published:** 2023-04-12

**Authors:** Man Cao, Jinhong Zhao, Cunrong Huang, Xianglin Wang, Lihong Ye, Xueyan Han, Wenzhou Yu, Zundong Yin, Juan Zhang, Yuanli Liu

**Affiliations:** ^1^School of Health Policy and Management, Chinese Academy of Medical Sciences & Peking Union Medical College, Beijing, China; ^2^Yale Graduate School of Arts and Sciences, New Haven, CT, United States; ^3^School of Population Medicine and Public Health, Chinese Academy of Medical Sciences & Peking Union Medical College, Beijing, China; ^4^National Immunization Program, Chinese Center for Disease Control and Prevention, Beijing, China

**Keywords:** vaccine hesitancy, confidence, validation, child, risks

## Abstract

**Introduction:**

Vaccine hesitancy may increase infectious disease burden and impede disease control efforts, while few studies have measured such a phenomenon with a standardized tool in China. This study aimed to test the validation of the Vaccine Hesitancy Scale (VHS) developed by the WHO SAGE Working Group among caregivers and examine demographic characteristics associated with caregiver hesitancy in six provinces of China.

**Methods:**

Using a multistage sampling design, this study was conducted in 36 immunization clinics in six provinces from December 2019 to August 2020. Caregivers of children aged 0–3  years were included. The VHS was used to assess vaccine hesitancy. The construct validity and internal consistency of the scale were assessed. Associations between caregivers’ characteristics and vaccine hesitancy were examined by simple and multiple linear regression models.

**Results:**

Of the 3,359 participants included, a two-factor structure within the scale was identified, consisting of “lack of confidence” (1.89 ± 0.53) and “risks” (3.20 ± 0.75). Caregivers engaged in medical work expressed more confidence and were less concerned about risks compared to those of non-medical staff (*p* < 0.05). Participants with higher income levels were more confident (*p* < 0.05), while those surveyed after the COVID-19 pandemic, who were mothers, who had an older child, or who were raising a second or above birth child, had less concern about risks (*p* < 0.05).

**Discussion:**

We found that the VHS had acceptable reliability and construct validity and caregivers’ hesitancy was driven more by concerns about risks than by the lack of confidence. Countering these concerns will be particularly important among non-medical staff, lower income, child’s fathers, having a younger child, or raising first-birth child groups.

## Introduction

It is well-recognized that immunization has profoundly contributed to the major decline in morbidity and mortality of particular infectious diseases (1, 2). Especially, the coronavirus disease-2019 (COVID-19) pandemic break out globally, and vaccination can be an effective strategy to protect public health (3). Surprisingly, surveys among people reveal that there is a significant rate of distrust against vaccines (4, 5). In addition, because of a wide range of dissemination of some pseudoscientific conclusions, for instance, MMR (Measles, mumps, and rubella combined vaccine) may cause autism (6), the public’s trust in vaccines has generally declined (7–9), triggering large-scale vaccine hesitancy. The vaccination rates for MMR in the United Kingdom sharply fell from 92% in 1995–1996 to 80% in 2003–2004 (6), and the United States and other countries have also been influenced to some extent (6, 10). Consequently, this situation led to outbreaks of vaccine-preventable diseases in some countries. To cite an example, from the end of 2014 to April 2015, two-thirds of measles outbreaks in the Americas were related to vaccine refusal (11, 12). Additionally, compared with high-income countries, middle- and low-income countries often have a larger population base and underdeveloped medical standards. Thus, once the diseases break out, the consequences will be disastrous.

In China, due to the frequent incidents on vaccine safety which caused panic about vaccination (13), Chinese researchers also pay more attention to vaccine hesitancy (14–18), and they found the illegal vaccine-selling events reduced caregivers’ trust in the vaccine, and some caregivers refused or hesitated to use vaccines for their children (19, 20). Nevertheless, there are some limitations in the existing research. The samples for these studies were only taken from one hospital or several hospitals in one city, which cannot represent the overall situation of China (14–17). Furthermore, the tools they used to evaluate vaccine hesitancy were different, including self-developed scales that lacked comparability among different studies, and some scales discussing vaccine hesitancy from only one dimension, which is not comprehensive enough (15, 17, 18). Moreover, some tools they used lacked the test of their reliability and validity which means that their reliability is questionable to some extent.

It is very vital to choose a suitable and standard research tool and to provide a standard framework to measure, evaluate, and compare vaccine hesitancy from different locations over time. The Vaccine Hesitancy Scale (VHS) was developed by the World Health Organization (WHO) Strategic Advisory Group of Experts (SAGE) Working Group to investigate vaccine hesitancy. It is a good tool with a uniform standard that has been applied to most countries in the world, making the results of the studies comparable with each other. Moreover, the VHS has moderate items and is not too complicated, thus participants may have a high degree of cooperation in the survey. In addition, it has been applied in many countries in the world, having potential value for international promotion (21–24). Furthermore, however, studies of vaccine hesitancy in China are few and limited in a standardized and validated measurement tool for international comparisons (25, 26).

Our study aimed to test the validation of the VHS scale in six provinces of China, measure caregivers’ vaccine hesitancy before and after the COVID-19 pandemic, and explore the influence of different characteristics of caregivers on vaccine hesitancy.

## Materials and methods

### Study design and participants

This cross-sectional study utilized a multistage sampling design. First, we purposefully selected seven representative provinces of China: three provinces, i.e., Shandong, Guangdong, and Zhejiang provinces in the eastern region, one in the central region (Henan Province), two in the western region (Sichuan Province and Inner Mongolia Autonomous Region), and one in the northeast region (Liaoning Province). Second, two prefecture-level cities were chosen from each province mentioned above, of which one city was in the upper 25th percentile of *per capita* GDP in the province, and the other was in the lower 25th percentile of *per capita* GDP. Third, one district or county with the 50th percentile of *per capita* GDP in each selected prefecture-level city was chosen. Fourth, healthcare facilities in the selected districts or counties were stratified by types (community healthcare centers, township clinics, and other medical and health institutions such as public hospitals or private health institutions); one immunization clinic was chosen on each stratum (if there was no other medical and health institution in the district or county, it would be replaced by the larger community health care center or township clinics). Within each immunization clinic, we selected a convenience sample of 90 caregivers. The survey was conducted from December 2019 to January 2020. Because of the outbreak of coronavirus disease-2019 (COVID-19) in January 2020, the investigation for two immunization clinics in Inner Mongolia Autonomous was postponed to be conducted from July to August 2020, and six immunization clinics in Zhejiang Province were withdrawn from the survey. Finally, the survey was conducted in 36 immunization clinics from 12 counties in six provinces.

Eligible participants included primary caregivers accompanying children aged 0–3 years who were born between 1 January 2017 and 1 January 2020 at immunization clinics in China. A total of 3,479 caregivers of children aged 0–3 years were invited to participate in the study, of which 3,474 caregivers agreed to participate, with a response rate of 99.86%. After excluding 63 duplicate coded questionnaires and 52 questionnaires with obvious logical errors, 3,359 caregivers of children were included in the data analysis.

### Data collection

**Figure 1:**
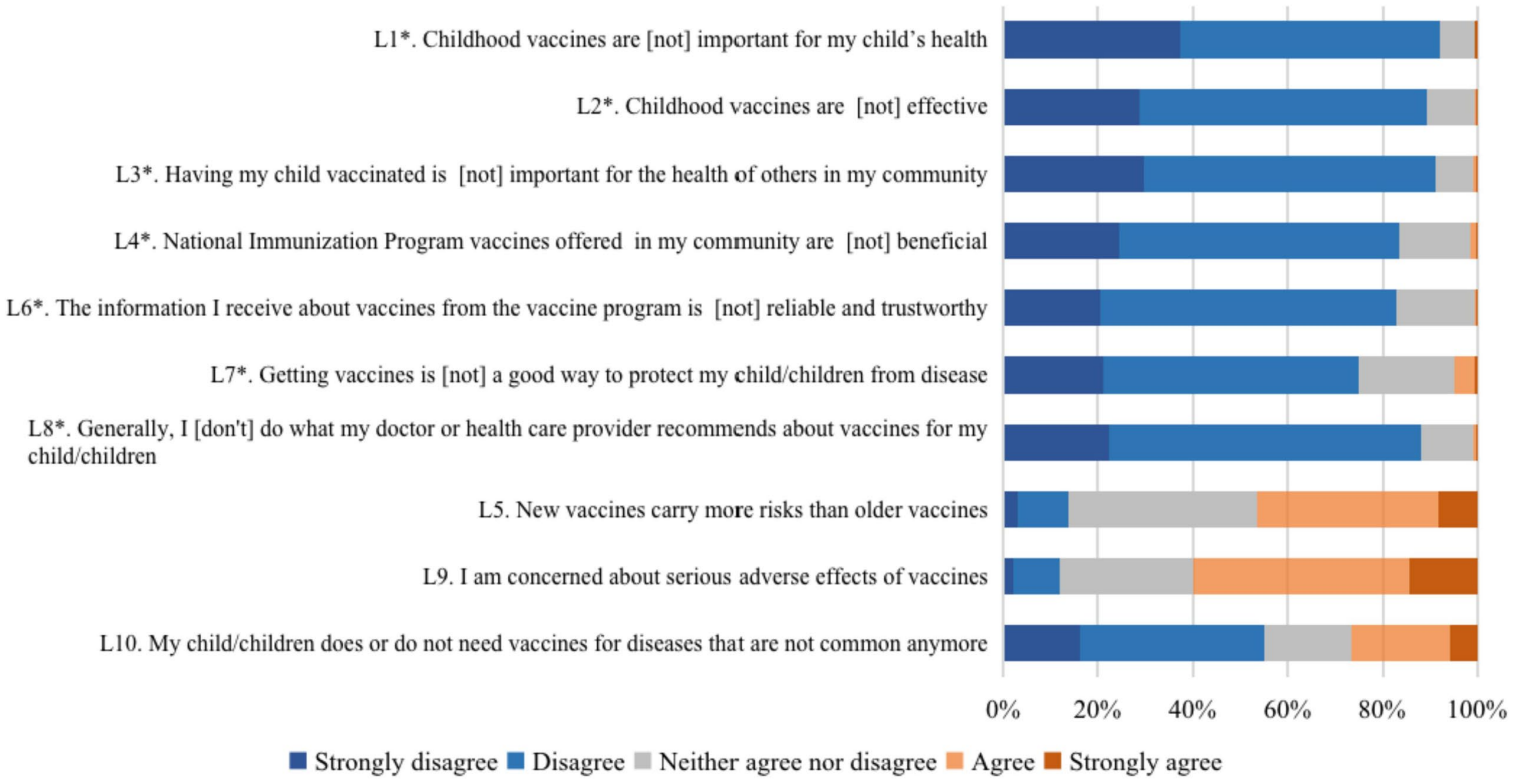
Distribution of responses to each item of the vaccine hesitancy scale. Items with a ^*^ were reverse coded.

Caregivers included were investigated through a face-to-face interview by trained interviewers. A structured questionnaire was designed to collect demographic information about children and their caregivers, such as children’s gender, age, birth order, the relationship between child and caregivers, caregivers’ age, education, occupation, and per-capita annual income. In addition, this study targets the vaccination of caregivers’ hesitancy in China, so we used 10 items of the VHS to assess vaccine hesitancy, in which each item was measured by a five-point Likert scale (1 = strongly disagree, 2 = disagree, 3 = neither agree nor disagree, 4 = agree, and 5 = strongly agree) (27). To make directionality uniform across all items, we reversely coded seven items (L1–L4, L6–L8), giving a higher score to disagreement than agreement, so that a higher score indicated more hesitancy on all items. The average of all items was calculated to assess the caregiver’s hesitancy for vaccination. All items for the VHS scale are described in Figure 1.

### Statistical analysis

The distribution of demographic characteristics and responses to the VHS scale was displayed using descriptive statistics. To analyze the construct validity of the scale, we conducted an exploratory factor analysis (EFA). Factors were extracted using a varimax or orthogonal rotation and an examination of Scree plots (28). We only retained the factors that had eigenvalues of at least 1.0. The reliability and internal consistency of the scale were assessed using Cronbach’s alpha (29). The association between demographic groups and components of the Vaccine Hesitancy Scale was assessed by simple and multiple linear regression models, with output *β* and 95% confidence intervals (*CI*). For multiple linear regression models, we used a backward stepwise method, using the criteria of *p* < 0.05 for inclusion and *p* > 0.10 for exclusion. For all analyses, a two-sided *p* value of <0.05 was considered significant, and all analyses were conducted using SAS (SAS 9.4, SAS Institute, Cary, NC, United States).

## Results

### Demographics of participants

The demographics for the final sample is presented in Table 1. The majority of the participants were mothers of children (72.13%) and most were 25–34 years old (65.56%). Around 70% of the participants had finished senior high school and 43.38% had a university education. Most participants were non-healthcare-related professionals (92.41%), with 7.59% of healthcare-related professions. Of the children surveyed, the majority were boys (51.59%), mostly concentrated in the age ≤ 12 months (53.77%), and most of them were firstborn (55.67%).

**Table 1:** Demographic characteristics of participants.

	*N*	Percentage
Total	3,359	100.00
Region
East	1,087	32.36
Center	638	18.99
West	1,083	32.24
Northeast	551	16.40
Period of survey
Before the COVID-19 pandemic	3,178	94.61
After the COVID-19 pandemic	181	5.39
Caregivers
Attainment of education
Junior high school or below	1,003	29.86
Senior high school	899	26.76
Bachelor degree	1,368	40.73
Master degree or above	89	2.65
Occupation
Non-healthcare-related profession	3,104	92.41
Healthcare-related profession	255	7.59
Age group (years)
<25	378	11.25
25–34	2,202	65.56
≥35	779	23.19
Per-capita annual income group (RMB)
<8,000	778	23.16
8,000–15,999	910	27.09
16,000–24,999	681	20.27
≥25,000	990	29.47
Relationship with child
Mother	2,423	72.13
Father	648	19.29
Grandparents	198	5.89
Other	90	2.68
Child		
Gender		
Boy	1,733	51.59
Girl	1,626	48.41
Age group (months)
≤12	1,806	53.77
13–24	945	28.13
25–36	608	18.10
The order of birth
First	1,870	55.67
Second or above	1,489	44.33

### Responses to vaccine hesitancy scale items

Most participants had positive beliefs about vaccination, while some participants expressed concerns about risks. Figure 1 shows parental responses to the 10 five-point Likert scale items of the WHO Vaccine Hesitancy Scale. Over 75% of participants showed positive attitude toward the seven positively phrased survey items (L1–L4, L6–L8). Notably, 91.96% of participants agreed or strongly agreed that “Childhood vaccines are important for my child’s health,” 91.22% reported “Having my child vaccinated is important for the health of others in my community,” and 89.28% reported, “Childhood vaccines are effective.” However, participants had less consistent responses to the 3 negatively phrased items (L5, L9, and L10), compared to the other seven positively phrased items. 59.53% of participants agreed that “I am concerned about serious adverse effects of vaccines,” 46.42% agreed that “New vaccines carry more risks than older vaccines,” and 26.71% believed that “My child does or does not need vaccines for diseases that are not common anymore.”

### Construct validity and internal consistency of the vaccine hesitancy scale

Exploratory factor analysis identified two factors with Eigenvalues higher than one (Table 2). These two factors explained 62.27% of the total variance of the 10-items scale, and one factor was predominant as it explained 45.07% of the total variance. Seven items were loaded on Factor 1, and they were primarily related to a lack of confidence in vaccines. Three items were loaded on Factor 2, and they were associated with vaccine risk and complacency as well as perceptions that vaccines are not beneficial. The Cronbach’s alpha value for the 10-item scale is equal to 0.80, indicating acceptable internal consistency reliability. Additionally, Cronbach’s alpha values were 0.91 and 0.62 for the “lack of confidence” factor and “risks” factor, respectively.

**Table 2:** Unrotated and rotated exploratory factor analysis factor loading pattern for the vaccine hesitancy scale items.

Vaccine hesitancy scale items	Factor pattern		Rotated factor pattern	
	VHS Factor 1: Lack of confidence	VHS Factor 2: Risks	VHS Factor 1: Lack of confidence	VHS Factor 2: Risks
L1*. Childhood vaccines are [not] important for my child’s health	**0.72256**	−0.10910	**0.71744**	−0.13888
L2*. Childhood vaccines are [not] effective	**0.78602**	0.14369	**0.79129**	0.11108
L3*. Having my child vaccinated is [not] important for the health of others in my community	**0.84635**	0.09351	**0.84950**	0.05845
L4*. National Immunization Program vaccines offered in my community are [not] beneficial	**0.83517**	0.06194	**0.83701**	0.02737
L6*. The information I receive about vaccines from the vaccine program is [not] reliable and trustworthy	**0.81637**	0.01077	**0.81612**	−0.02298
L7*. Getting vaccines is [not] a good way to protect my child/children from disease	**0.78834**	−0.04483	**0.78581**	−0.07738
L8*. Generally, I [do not] do what my doctor or health care provider recommends about vaccines for my child/children	**0.79238**	−0.04504	**0.78984**	−0.07776
L5. New vaccines carry more risks than older vaccines	−0.13846	**0.73321**	−0.10803	**0.73830**
L9. I am concerned about serious adverse effects of vaccines	0.09355	**0.75242**	0.12457	**0.74791**
L10. My child/children does or do not need vaccines for diseases that are not common anymore	−0.09507	**0.75332**	−0.06385	**0.75661**

Items with a * were reverse coded. VHS, vaccine hesitancy scale.

### Demographic variables associated with vaccine hesitancy

There was a greater endorsement of the “risks” factor (3.20 ± 0.75) compared to the “lack of confidence” factor (1.89 ± 0.53). Simple and multiple linear regression model results for two factors of vaccine hesitancy are shown in Table 3. The occupation was a significant predictor for both two factors. Compared to caregivers who were in a non-healthcare-related profession, caregivers engaged in the healthcare-related profession expressed more confidence (*β*: -0.15, 95% *CI*: −0.22 to −0.08) and were less concerned about risks (*β*: -0.10, 95% *CI*: −0.19 to −0.00). For the “lack of confidence” factor, participants with higher per-capita annual income levels were more confident with vaccine than counterparts with lower income (16,000–24,999 vs. <8,000RMB *β*: −0.11, 95% *CI*: −0.17 to −0.06; ≥25,000 vs. <8,000RMB *β*: −0.10, 95% *CI*: −0.15 to −0.05). Grandparents had more confidence than fathers (*β*: −0.11, 95% *CI*: −0.19 to −0.03). For the “risk” factor, caregivers surveyed after the COVID-19 pandemic expressed less concern about risks (*β*: −0.16, 95% *CI*: −0.28 to −0.04). Compared to fathers, mothers were less concerned about risks (*β*: −0.12, 95% *CI*: −0.18 to −0.05). Participants who had an older child or raised a second or above birth child had less concern about risks (*β*: −0.08, 95% *CI*: −0.13 to −0.03).

**Table 3:** Demographic characteristics and their relation to two components of vaccine hesitancy.

	VHS Factor 1: Lack of confidence					VHS Factor 2: Risks				
	Mean (SD)	Simple linear regression		Multiple linear regression		Mean(SD)	Simple linear regression		Multiple linear regression	
		*β* (95% *CI*)	*p*	*β* (95% *CI*)	*p*		*β* (95% *CI*)	*p*	*β* (95% *CI*)	*p*
Total	1.89 (0.53)					3.20 (0.75)				
Region
East	1.88 (0.52)	−0.10 (−0.14, −0.05)	<0.001	−0.10 (−0.14, −0.05)	<0.001	3.14 (0.76)	−0.04 (−0.11, 0.02)	0.166	−0.06 (−0.13, 0.00)	0.066
Center	1.82 (0.53)	−0.16 (−0.21, −0.11)	<0.001	−0.18 (−0.23, −0.12)	<0.001	3.18 (0.80)	−0.00 (−0.08, 0.07)	0.911	−0.02 (−0.10, 0.05)	0.571
West	1.98 (0.50)	Reference		Reference		3.19 (0.67)	Reference		Reference	
Northeast	1.86 (0.55)	−0.12 (−0.17, −0.06)	<0.001	−0.10 (−0.16, −0.05)	<0.001	3.34 (0.77)	0.15 (0.07, 0.22)	0.000	0.12 (0.04, 0.20)	0.003
Period of survey
Before the COVID-19 pandemic	1.89 (0.53)	Reference				3.21 (0.75)	Reference		Reference	
After the COVID-19 pandemic	1.98 (0.41)	0.09 (0.01, 0.17)	0.021	-	-	3.04 (0.66)	−0.16 (−0.28, −0.05)	0.004	−0.16 (−0.28, −0.04)	0.009
Attainment of education
Junior high school or below	1.92 (0.53)	Reference				3.17 (0.74)	Reference			
Senior high school	1.92 (0.54)	−0.00 (−0.05, 0.05)	0.941	-	-	3.19 (0.75)	0.02 (−0.05, 0.09)	0.569	-	-
Bachelor degree	1.86 (0.52)	−0.06 (−0.11, −0.02)	0.003	-	-	3.21 (0.75)	0.04 (−0.03, 0.10)	0.256	-	-
Master degree or above	1.83 (0.49)	−0.10 (−0.21, 0.02)	0.094	-	-	3.28 (0.73)	0.10 (−0.06, 0.26)	0.215	-	-
Occupation
Non-healthcare-related profession	1.91 (0.53)	Reference		Reference		3.21 (0.74)	Reference		Reference	
Healthcare-related profession	1.75 (0.51)	−0.16 (−0.23, −0.09)	<0.001	−0.15 (−0.22, −0.08)	<0.001	3.08 (0.84)	−0.12 (−0.22, −0.03)	0.011	−0.10 (−0.19, −0.00)	0.048
Age group (years)
<25	1.95 (0.49)	Reference				3.15 (0.70)	Reference			
25–34	1.89 (0.53)	−0.06 (−0.12, −0.00)	0.039	-	-	3.20 (0.75)	0.05 (−0.03, 0.14)	0.192	-	-
≥35	1.87 (0.52)	−0.08 (−0.15, −0.02)	0.011	-	-	3.20 (0.75)	0.05 (−0.04, 0.15)	0.246	-	-
Per-capita annual income group (RMB)
<8,000	1.94 (0.54)	Reference		Reference		3.15 (0.75)	Reference			
8,000–15,999	1.94 (0.50)	0.00 (−0.05, 0.05)	0.948	−0.00 (−0.06, 0.04)	0.793	3.21 (0.73)	0.05 (−0.02, 0.12)	0.154	-	-
16,000–24,999	1.84 (0.52)	−0.10 (−0.16, −0.05)	0.000	−0.11 (−0.17, −0.06)	<0.001	3.23 (0.77)	0.07 (−0.01, 0.15)	0.070	-	-
≥25,000	1.85 (0.54)	−0.09 (−0.14, −0.04)	0.000	−0.10 (−0.15, −0.05)	<0.001	3.20 (0.74)	0.05 (−0.02, 0.12)	0.185	-	-
Relationship with child
Mother	1.90 (0.51)	0.00 (−0.04, 0.05)	0.942	0.01 (−0.04, 0.05)	0.733	3.17 (0.72)	−0.12 (−0.19, −0.06)	0.000	−0.12 (−0.18, −0.05)	0.000
Father	1.90 (0.58)	Reference		Reference		3.29 (0.79)	Reference		Reference	
Grandparents	1.83 (0.49)	−0.07 (−0.15, 0.01)	0.100	−0.11 (−0.19, −0.03)	0.010	3.27 (0.76)	−0.01 (−0.13, 0.10)	0.819	0.00 (−0.12, 0.12)	0.953
Other	1.80 (0.68)	−0.10 (−0.21, 0.02)	0.107	−0.11 (−0.23, 0.00)	0.057	3.24 (0.88)	−0.05 (−0.21, 0.11)	0.554	−0.03 (−0.20, 0.13)	0.696
Child
Gender										
Boy	1.89 (0.53)	Reference				3.18 (0.76)	Reference			
Girl	1.9 (0.52)	0.02 (−0.02, 0.05)	0.298	-	-	3.22 (0.73)	0.04 (−0.02, 0.09)	0.171	-	-
Age group (months)
≤12	1.9 (0.53)	Reference				3.23 (0.74)	Reference		Reference	
13–24	1.91 (0.52)	0.00 (−0.04, 0.05)	0.810	-	-	3.18 (0.73)	−0.05 (−0.11, 0.016)	0.103	−0.06 (−0.12, 0.00)	0.058
25–36	1.86 (0.52)	−0.04 (−0.09, 0.00)	0.074	-	-	3.12 (0.76)	−0.11 (−0.18, −0.04)	0.001	−0.11 (−0.18, −0.05)	0.001
The order of birth
First	1.89 (0.52)	Reference				3.25 (0.73)	Reference		Reference	
Second or above	1.9 (0.53)	0.00 (−0.03, 0.04)	0.865	-	-	3.13 (0.76)	−0.11 (−0.16, −0.06)	<0.001	−0.08 (−0.13, −0.03)	0.003

VHS, vaccine hesitancy scale; SD, standard deviation.

## Discussion

Understanding vaccine hesitancy has become a priority in China, especially with the spread of misinformation surrounding the ongoing pandemic COVID-19 and a series of “vaccination crises” amplifying vaccine hesitancy over the last decade (30–34). To the best of our knowledge, this study is the first large-scale study to report the level of vaccine hesitancy among caregivers of children under 3 years old in 36 immunization clinics from 12 counties in six provinces before and after the COVID-19 pandemic, using the validated Vaccine Hesitancy Scale developed by the WHO. The scale consists of two factors: lack of confidence and risks, and shows acceptable reliability and validity in China. The present study suggests that caregivers with a non-healthcare-related profession, lower per-capita annual income, and who are a father, raising a younger child, and raising the first child, have a high level of vaccine hesitancy.

Our study revealed that the VHS scale with a two-factor structure existed acceptable validity and reliability among Chinese caregivers. Moreover, the scale has been widely used in much different literature contexts (21–24, 35–43). Masters et al. (35) and Wagner et al. (36) used the 10-item VHS to assess vaccine hesitancy among caregivers in Ethiopia and India, respectively. Kim et al. (39) examined the hesitancy of nurses on human papillomavirus vaccinations with the scale in Korea. Modifications of the VHS were made to better adapt to contexts in prior studies. On the other hand, some studies showed that modifications of the VHS were made to better adapt to the contexts. For instance, some researchers found that item 10, “My child does or does not need vaccines for diseases that are not common anymore,” did not agree with the other factors and was thus excluded from final analyses in America, Britain, and Canada (22, 24, 40). Because most vaccination is not national program to provide free vaccination in the United States, item 4, “All childhood vaccines offered by the government program in my community are beneficial,” was eliminated by Szilagyi et al. (42) and Kempe et al. (43). In another two surveys, the item was modified to “All childhood vaccines offered by my child’s healthcare provider are beneficial” (40) and “All routine vaccinations recommended by the CDC are beneficial” (21), respectively. A study found the EFA model was best fit with a seven-item scale (without item 3, item 6, and item 9) rather than a 10-item scale in Guatemala (23). We also found that the VHS scale with the deletion of item 10 produced higher internal consistency (Cronbach’s alpha = 0.81) in our study, while the internal consistency for the “risks” factor declined (Cronbach’s alpha = 0.55) so all 10 items were included in the analyses (Cronbach’s alpha = 0.80). Accordingly, the VHS could be used as a validated standardization tool to measure vaccine hesitancy among children’s caregivers.

The COVID-19 pandemic can be considered a global unifier, with countries worldwide all challenged to contain the spread of novel coronavirus (44). In our study, we found participants surveyed after the pandemic, from July to August 2020, reported less hesitancy in vaccines, especially for concerns about risks, compared to those surveyed before the pandemic. These findings implied caregivers’ expectations for vaccination. Globally, in March 2020, the average vaccine acceptance observed was 86% which dropped to 54% in July 2020 and later increased to 72% in September 2020 (45). It can be seen that people’s willingness, acceptance, and trust in vaccines were relatively high in the early stage of COVID-19, and then gradually declined.

We found that vaccine hesitancy was driven more by risk perceptions than by a lack of confidence among Chinese caregivers. This result was consistent with many previous surveys in other countries and caregivers expressed more hesitancy about risks, especially in China (46, 47). Hesitancy scores on risks in this study (3.20 ± 0.75) were higher than in Canada (24) (3.07 ± 0.95), Britain (22) (2.89 ± 0.93), and India (36) (2.84 ± 0.68). For hesitancy scores on lack of confidence, it was a little lower in this study (1.89 ± 0.53) than in Canada (1.98 ± 0.72) and Britain (1.99 ± 0.80), but higher than in India (1.63 ± 0.35). These findings suggested that caregivers may perceive risks in China, compared to other countries. As highlighted in this study, health education on vaccination risk should be emphasized to raise public knowledge and understand risks better, especially the serious adverse effects of vaccines. Additionally, our results may provide a basic reference for subsequent vaccine hesitancy-related research in China.

Vaccine hesitancy could be associated with a variety of sociodemographic factors. We found that caregivers who were in healthcare-related profession had more confidence and were less concerned about risks compared to those in the non-healthcare-related profession. Possible reasons may include a better understanding of vaccination. Healthcare-related profession have gained more information and knowledge about vaccination, and are more likely to recognize the importance and effectiveness of vaccination (25). Moreover, our results from a simple linear regression model showed caregivers with higher education had more confidence, similar to studies in India (36), Canada (24), America (43), and Shanghai, China (37). Although there is rising concern globally that higher educated groups are more likely to seek exemptions (48–50) or to express safety concerns (51), this is not a uniform pattern (26). A study did not find a significant impact of education level on vaccine hesitancy across five Low- and Middle-Income Countries (LMICs) (38), and we also did not find a significant association between education levels with vaccine hesitancy in a multiple linear regression model. Our study showed that caregivers with higher per-capita annual income were associated with more confidence in vaccination, consistent with the findings of some previous studies (24, 43). However, other studies reported mixed results concerning the relationship between income and attitude toward vaccination (50, 52, 53). For example, Opel et al. (52) found that caregivers with higher income were two times more likely to be concerned about serious vaccine-related adverse reactions than counterparts with lower income. We observed different associations compared to a Canadian study (24), in that our study showed fathers have heightened concern about certain vaccine risks compared to mothers. Moreover, another study reported that fathers expressed greater beliefs that new vaccines were riskier than older ones in Shanghai, China (37). Furthermore, we found caregivers having younger or first-birth child revealed more concerns about risks. Nevertheless, previous studies had shown no significant association (35, 36). Counseling this group might be effective, reasons behind this may be due to caregivers’ experience and attention. Caregivers may lack experience in taking care of a young or first-birth child, and they were more likely to feel more concerned about vaccination, especially its risks. These additional findings help aid further research and development of strategies to drive vaccine acceptance. Therefore, it is recommended that China’s healthcare department should pay attention to the phenomenon of vaccine hesitancy and raise the awareness of parents of children about the benefits of vaccination. In addition, they should learn from the experience of international countries to solve the problem of vaccine hesitancy in China (54, 55), such as improving the vaccine market access mechanism and standardizing the vaccination process to reduce the occurrence of adverse safety events.

This study has several limitations. First, caregivers’ hesitancy may be possibly affected by other factors, such as the experience of vaccination service, the type of vaccine, and the manufacturer of vaccine. Future studies should consider these factors. Second, we did not collect information on which vaccine the child was supposed to get when they got to the clinic, so it was limited to knowing if this hesitancy was associated with the type of vaccine they were getting. Third, most of the participants were mothers of children, which may lead to missing fathers’ perceptions about vaccination. Fourth, few items loaded on the “risks” component and a lack of positively and negatively worded items for both components.

## Conclusion

Our findings underscored that the Vaccine Hesitancy Scale may be served as a valid and reliable tool for assessing vaccine hesitancy to provide formulation and standardization measurement instruments in future investigations. We found the scale consisted of two factors, including “lack of confidence” and “risks.” The caregiver’s hesitancy was driven more by concerns about risks than by a lack of confidence. Countering these concerns will be particularly important in non-healthcare-related profession, lower income, child’s fathers, and having younger or first-birth child groups. Future research is needed to explore more possible determiners of caregiver vaccine hesitancy with the scale to guide educational and outreach strategies in China.

## Data availability statement

The original contributions presented in the study are included in the article/supplementary material, further inquiries can be directed to the corresponding authors.

## Ethics statement

The studies involving human participants were reviewed and approved by Chinese Center for Disease Control and Prevention Institutional Review Board (#201944). The patients/participants provided their written informed consent to participate in this study.

## Author contributions

MC, JuZ, YL, WY, and ZY designed the study. MC, JiZ, CH, LY, and XW collected the data from 36 immunization clinics in China. MC, CH, and JiZ reviewed the literature, performed the analyses, and wrote the first draft of the manuscript. JuZ, YL, and XH critically revised the manuscript. All authors contributed to the article and approved the submitted version.

## Funding

This study was funded by the China Medical Board (Grant Number 20–379) and the Chinese Academy of Medical Sciences (CAMS) Innovation Fund for Medical Sciences (2021-I2M-1-046).

## Conflict of interest

The authors declare that the research was conducted in the absence of any commercial or financial relationships that could be construed as a potential conflict of interest.

## Publisher’s note

All claims expressed in this article are solely those of the authors and do not necessarily represent those of their affiliated organizations, or those of the publisher, the editors and the reviewers. Any product that may be evaluated in this article, or claim that may be made by its manufacturer, is not guaranteed or endorsed by the publisher.
